# Abscess of the Antrum of Highmore Simulating Acute Inflammation of the Lachrymal Sac

**Published:** 1881-03

**Authors:** B. H. Grove


### Clinical Reports.

### ABSCESS OF THE ANTRUM OF HIGHMORE SIMULATING ACUTE INFLAMMATION OF THE LACHRYMAL SAC.

REPORTED BY B. H. GROVE, M. D., FROM THE PRACTICE OF DR. LUCIEN HOWE.

THE differential diagnosis in the following case, a certain peculiarity in the symptoms presented, and the method of treatment employed, seem to make it worthy of some notice.

The patient, J. C., was a robust fellow, 27 years old, who applied for relief at the Buffalo Eye and Ear Infirmary on the 21st of last August.

The history of his case, as detailed in rather an intelligent manner, was about as follows: Some four months previously he first noticed a swelling just below the left eye. He could not say whether this was near the outer or the inner angle of the lid, but simply that it was in that region. Inflammatory symptoms gradually increased, however, pain being a very prominent one, and in the course of a couple of weeks a few drops of pus were evacuated through an opening, which formed near the centre of the lower lid and about half an inch below the margin. Temporary improvement followed, but when this opening closed, the same train of symptoms reappeared, another fistulous opening formed near the first and considerable pus discharged through the nostril on that side. When the patient applied for relief, as above stated, the following condition presented. The tissues about the lower part of the left eye were considerably swollen, the concavity between the nose and eye being almost entirely filled out, and the enlargement extending almost out to the outer angle of the lid. Near its centre there was a dull red spot, marking the position of the first opening, and a little internal to this, a fistula, through which pus exuded freely. The nostril on that side was entirely closed and the patient said there was a considerable accumulation of pus—not of mucous—in the throat every morning. The epiphora was decided, but not profuse; pain was usually severe, but varied.

Dr. Howe prescribed a weak solution of sulphate of zinc—grs. ii ad ℥ i—and instructed the patient to inject into the opening, by means of a small pipette, first, a generous quantity of tepid water, and then a few drops of this astringent solution. That was to be done once or twice a day, at least. Directions were also given to inject into the nostril on that side, a weak solution of potassa permanganate, and an anodyne mixture was given to be applied locally in case of pain. Before Sept. 8th the discharge had lessened considerably, and the patient felt much better. On the 16th of that month, a piece of bone measuring about ¼x½ an inch was removed from the nostril, and the patient said that "other pieces like that"—perhaps one or two—"had come away." On the 28th the fistula had entirely closed, and on Oct. 9th the man was discharged. The situation of the opening marked by an adherent cicatrix about a quarter of an inch in diameter, which produced a slight deformity, but the eye and lid were otherwise in excellent condition.

In the history of this case, there are at least three points which seem to be worthy of attention. They relate to the diagnosis, the position of the openings and the method of treatment. Very few works on diseases of the eye mention an abscess in the antrum as similar to a dachryocystitis. Schirmer,* refers indirectly to the possibility of such a condition, but most writers ignore the fact as thus exemplified, that the two diseases are liable to be confused.

We have here, however, a tumefied condition of the entire lower lid, together with pain and tenderness. The first and second openings might have been ascribed to an accumulation of pus in the lachrymal sac which had burrowed under the tissues before finding vent, and the entire closure of the nostril would have corroborated that view of the case It is true the absence or presence of a carious tooth, and the duration of the complaint were to be considered as important evidence, in arriving at the conclusion, but altogether the diagnosis in the case could not be regarded as entirely free from difficulty.

A second point noticeable in the history of this case is the place of perforation of the abscess. A portion of the pus of course found exit through the nostril, but it is very seldom that the hard part of the superior maxillary bone, which forms the lower border of the orbit, is so far absorbed as to allow a fluid to pass through it. At least, I find no such case recorded.

Finally the treatment, while not so severe as that usually adopted of puncturing the bone in the vicinity of the canine fossa or elsewhere, or even of the extraction of a tooth, was, after all quite effectual. It is true an opening into the nose had already been formed in addition to the one above, and it is also probable that only in such exceptional cases would the plan adopted by Dr. Howe, be found advisable. We must, however, admit that the simple washing out of the cavity, first with water and afterwards with a mild astringent solution,

*Graefe & Saemisch, Handbuch der Augenheilkunde, Band VII.

is preferable to operative procedures wherever admissible. It is another illustration of the fact that we will be better surgeons when we use instruments less if at all, and any advance in this direction, however slight, seems worthy of imitation.

